# Conservative deflationism?

**DOI:** 10.1007/s11098-018-1193-5

**Published:** 2018-11-14

**Authors:** Julien Murzi, Lorenzo Rossi

**Affiliations:** 1 Philosophy Department (KGW), University of Salzburg, Salzburg, Austria; 2 Munich Centre for Mathematical Philosophy, Ludwig-Maximilians University, Munich, Germany

**Keywords:** Truth, Deflationism, Conservativeness, Categoricity, Reflection principles, Isaacson's thesis

## Abstract

Deflationists argue that ‘true’ is merely a logico-linguistic device for expressing blind ascriptions and infinite generalisations. For this reason, some authors have argued that deflationary truth must be *conservative*, i.e. that a deflationary theory of truth for a theory *S* (that interprets a sufficient amount of mathematics, or syntax) must not entail sentences in *S*’s language that are not already entailed by *S*. However, it has been forcefully argued that any adequate theory of truth for *S* must be non-conservative and that, for this reason, truth cannot be deflationary (Shapiro in J Philos XCVI(10):493–521, 1998; Ketland in Mind 108(429):69–94, 1999). We consider two defences of conservative deflationism, respectively proposed by Waxman (Mind 126(502):429–463, 2017) and Tennant (Mind 111(443):551–582, 2002), and argue that they are both unsuccessful. In Waxman’s hands, deflationists are committed either to a non-purely expressive notion of truth, or to a conception of mathematics that does not allow them to justifiably exclude non-conservative theories of truth. Tennant’s conservative deflationism fares no better: if deflationist truth must be conservative over arithmetic, it can be shown to collapse into a non-conservative variety of deflationism.

Deflationism about truth has it that ‘true’ is a logico-linguistic device for expressing blind ascriptions and infinite generalisations (as in ‘All of Peano Arithmetic’s theorems are true’). As Vann McGee puts it, according to deflationists truth ‘doesn’t have any legitimate applications beyond its logical uses, so it cannot play a significant theoretical role in scientific inquiry or causal explanation’ (McGee 2016, p. 3153).^1^ Some authors go further and argue that a theory of truth for a theory *S* ought to yield a conservative extension of *S*, i.e. it shouldn’t entail sentences in the truth-free language that aren’t already entailed by *S*.^2^ In a slogan, deflationary truth must be conservative.

However, Stewart Shapiro (1998) and Jeffrey Ketland (1999) have long argued that any minimally adequate theory of truth cannot be conservative. A theory of truth for first-order Peano Arithmetic (PA), their argument goes, must allow one to prove sentences such as $G_{\mathsf {PA}}$, the Gödel sentence for PA, and Con(PA), a sentence expressing PA’s consistency.^3^ But neither $G_{\mathsf {PA}}$ nor Con(PA) are entailed by PA, if PA is consistent. They conclude that truth cannot be deflationary, since it allows one to prove substantive arithmetical truths. Call this the *conservativeness argument*.

In a recent paper, Waxman (2017) observes that the conservativeness requirement is ambiguous between two readings, a semantic and a syntactic one, corresponding to two different conceptions of arithmetic. On the first reading, arithmetic is understood categorically, i.e. as given by the standard model ${\mathbb {N}}$. On the second reading, arithmetic is understood axiomatically, i.e. as exhausted by the acceptance of some non-categorical (typically first-order) theory such as PA. According to Waxman, deflationary truth can be conservative on either reading and the conservativeness argument does not go through. More precisely, on the categorical conception, every (consistent) theory of truth is semantically conservative over its arithmetical base theory; on the syntactic conception, sentences such as $G_{\mathsf {PA}}$ and Con(PA) are not part of one’s conception of arithmetic, whence one *ought not* to be able to prove them in the first place. Either way, Waxman maintains, deflationary truth can be conservative.

We argue that Waxman’s defence of deflationary but conservative truth is unsuccessful. By our lights, the semantic horn of Waxman’s argument has already been shown to be problematic. Waxman’s categorical deflationists resort to a notion of truth-in-a-higher-order-structure that appears to be in tension with the purely expressive deflationary conception of truth (Shapiro 1998; Cieśliński 2015).^4^ Accordingly, we mostly focus on the syntactic horn. We suggest that the axiomatic conception of arithmetic is both unduly restrictive and self-undermining: it excludes standard pieces of mathematical knowledge such as $G_{\mathsf {PA}}$ and Con(PA), and it does not even allow the axiomatic deflationist to coherently justify her refusal to accept such sentences. Along the way, we suggest that a strategy advocated by Tennant (2002) for escaping the conservativeness argument while at the same time retaining knowledge of $G_{\mathsf {PA}}$ and Con(PA) is also problematic. Assuming with Tennant that an acceptable deflationary theory of truth over PA must be conservative over PA, we notice that the deflationist who wishes to recover standard mathematical knowledge must also accept theories of arithmetic in which a non-conservative truth predicate can be defined.

While our arguments do not tell against deflationism in general, they suggest that there is little conceptual space, if any, for conservative deflationism, especially if one’s conception of mathematics is axiomatic, i.e. exhausted by one’s acceptance of the axioms of a first-order theory such as PA. The idea that our grasp of mathematical notions is given by our acceptance of axiomatic theories is both appealing and persistent.^5^ Our arguments show that, on such a conception, irrespective of which mathematical axioms are accepted, one cannot reject non-conservative theories of truth on the grounds that they are not conservative. More precisely, conservative deflationists whose conception of mathematics is axiomatic cannot reject mathematical claims on the grounds that they don’t follow from the axioms of their theory. We take this to be evidence that the deflationist’s commitment to a conservative conception of truth is misguided.^6^

The remainder of the paper is organised as follows. Section 1 sets up the scene. Section 2 presents Waxman’s disjunctive argument. Sections 3–5 articulate our responses to Waxman and Tennant. Section 6 concludes.

## Technical preliminaries

Following Waxman, we define the following two notions of conservativeness of a theory $S^+$ with language ${\mathcal {L}}_{S^+}$ over a theory *S* with language ${\mathcal {L}}_S$, where ${\mathcal {L}}_S \subseteq {\mathcal {L}}_{S^+}$:

### **Definition 1**

(*Syntactic conservativeness*) $S^+$ is a syntactic conservative extension of *S* if, for every $\varphi \in {\mathcal {L}}_S$, if $S^+ \vdash \varphi$, then $S \vdash \varphi$.

### **Definition 2**

(*Semantic conservativeness*) $S^+$ is a semantic conservative extension of *S* if, for every $\varphi \in {\mathcal {L}}_S$, if $S^+\, \vDash\, \varphi$, then $S \,\vDash\, \varphi$.

These two notions are coextensive if the consequence relation expressed by $\vDash$ is complete; they may come apart otherwise (for instance, completeness fails for second-order consequence).

Still following Waxman, we let (first-order) PA be our base theory, i.e. the theory to which we add the principles governing the truth predicate, and ${\mathcal {L}}_{\mathsf {PA}}$ be its language. PA contains axioms for the basic arithmetical functions and operations (successor, addition, multiplication and exponentiation), as well as every instance of the induction schema

$$\begin{aligned}&({\mathsf {Induction}}\,\,{\mathsf {Schema}}) \quad \varphi (0) \wedge \forall \;x (\varphi (x) \rightarrow \varphi ({\mathsf {S}}(x))) \rightarrow \forall \;x\varphi (x), \end{aligned}$$

for every formula $\varphi \in {\mathcal {L}}_{{\mathsf {PA}}}$, where ${\mathsf {S}}(x)$ is the successor function applied to *x*. In order to add a theory of truth to PA, we need to expand ${\mathcal {L}}_{{\mathsf {PA}}}$ to a richer language ${\mathcal {L}}_{{\mathsf { PA}}}^+$, given by ${\mathcal {L}}_{\mathsf {PA}} \cup \{{\mathsf {Tr}}\}$, where Tr is a one-place predicate expressing truth. Following Tarski (1956), we assume that, for some appropriate coding scheme, the theory of truth for PA is materially adequate, i.e. that it derives all instances of the T-schema

$$\begin{aligned}&({\mathsf {T}}\text{- }{\mathsf {Schema}}) \qquad {\mathsf {Tr}}(\ulcorner \varphi \urcorner ) \leftrightarrow \varphi , \end{aligned}$$

where $\varphi \in {\mathcal {L}}_{\mathsf {PA}}$ and $\ulcorner \varphi \urcorner$ is the numeral of the code of $\varphi$. Still following Tarski, we also assume that an adequate theory of truth must prove ‘the most important and most fruitful general theorems’ Tarski (1956, p. 257), such as that every sentence is either true or untrue, that no sentence is both true and untrue, and so on. Since the T-schema only proves every instance of such theorems but not the general claims, a theory of truth must therefore include the Tarskian compositional clauses for the logical operators and predicates of the base language—for instance, that a conjunction is true if and only if both of its conjuncts are true, that a universally quantified sentence $\forall x\varphi$ is true if and only if each of its instances $\varphi [t/x]$ is true, and so on.^7^

We can distinguish between two versions of the compositional theory: ${\mathsf {PA}}_{\mathsf {Tr}}$, which is given by PA together with the compositional axioms for ${\mathsf {Tr}}$; and ${\mathsf {PA}}_{\mathsf {Tr}}^+$, which is given by adding to ${\mathsf {PA}}_{\mathsf {Tr}}$ all the instances of the Induction Schema that in which the truth predicate occurs.^8^ It is well-known that that while ${\mathsf {PA}}_{\mathsf {Tr}}$ is syntactically conservative over PA, ${\mathsf {PA}}_{\mathsf {Tr}}^+$ isn’t. In particular, the so-called *semantic argument*, an argument establishing the consistency of PA, can be carried out in ${\mathsf {PA}}_{\mathsf {Tr}}^+$. The argument proceeds as follows. First, one observes in ${\mathsf {PA}}_{\mathsf {Tr}}^+$ that all of PA’s axioms are true and that all of PA’s inference rules preserve truth. Second, one infers, reasoning in ${\mathsf {PA}}_{\mathsf {Tr}}^+$, that all of PA’s theorems are true. Third, one notices that ${\mathsf {PA}}_{\mathsf {Tr}}^+$ proves $\lnot {\mathsf {Tr}}(\ulcorner 0=1\urcorner )$, and hence that $0=1$ is not a theorem of ${\mathsf {PA}}$, since ${\mathsf {PA}}_{\mathsf {Tr}}^+$ proves that every theorem of ${\mathsf {PA}}$ is true. But the sentence ‘$0=1$ is not a theorem of ${\mathsf {PA}}$’ is formalized in ${\mathcal {L}}_{\mathsf {PA}}$ as ${\mathsf {Con(PA)}}$, the canonical consistency statement for ${\mathsf {PA}}$. So, ${\mathsf {PA}}_{\mathsf {Tr}}^+$ proves the consistency of PA, which by Gödel’s Second Incompleteness Theorem is *not* provable in PA itself, if PA is consistent. Moreover, since $G_{\mathsf {PA}}$ is provably equivalent to ${\mathsf {Con(PA)}}$ in PA, the semantic argument also establishes that ${\mathsf {PA}}_{\mathsf {Tr}}^+$ proves $G_{\mathsf {PA}}$. Crucially, the semantic argument requires that the Induction Schema be extended to formulas containing ${\mathsf {Tr}}$, and therefore cannot be carried out in ${\mathsf {PA}}_{\mathsf {Tr}}$.

Critics of deflationism, such as Ketland and Shapiro, maintain that ‘in one form or another, conservativeness is essential to deflationism’ (Shapiro 1998, p. 497). At the same time, they argue that any adequate theory of truth must be able to reproduce the semantic argument. Insofar as conservativeness is interpreted in syntactic terms, it follows that deflationary theories of truth cannot be adequate. As Ketland puts it,

our ability to recognize the truth of Gödel sentences involves a theory of truth ...which *significantly transcends the deflationary theories*. (Ketland 1999, p. 88)

Waxman disagrees. In his view, deflationary truth can be both conservative and adequate.

## Waxman’s disjunctive argument

Waxman begins by observing that the conservativeness requirement is ambiguous between a syntactic and a semantic reading. He then considers two broad families of conceptions of arithmetic. As he puts it,

[t]he first conception is broadly model-theoretic in nature: for lack of a better term, let us call it the *categorical conception* of arithmetic. It holds that arithmetic is a subject about a particular mathematical structure—the natural numbers, $[{\mathbb {N}}]$, the elements of which are obtained by beginning with 0 and iterating the successor operation finitely many times. By contrast, the other conception is broadly proof-theoretic in nature: let us call it the *axiomatic conception* of arithmetic. It holds that our best understanding of arithmetic consists in (and is exhausted by) the proof-theoretic consequences of a particular set of axioms, namely, first-order PA. (Waxman 2017, p. 447; emphases added)

On the categorical conception, conservativeness is defined in semantic terms; on the axiomatic conception, conservativeness is defined in syntactic terms. Waxman contends that, irrespective of how arithmetic is understood, the conservativeness argument breaks down.

Consider the first case first, i.e. suppose the deflationist’s conception of arithmetic is given by a categorical theory of arithmetic, such as second-order Peano Arithmetic (${\mathsf {PA}}_{\mathsf {2}}$). ${\mathsf {PA}}_{\mathsf {2}}$ is given by the axioms of PA formulated in second-order logic, a comprehension schema for any formula of the language, and with the infinitely many instances of the Induction Schema replaced by the second-order Induction Axiom:

$$\begin{aligned}&({\mathsf {Induction}}\, {\mathsf {Axiom}}) \quad \forall X [X(0) \wedge \forall \; x (X(x) \rightarrow X(Sx)) \rightarrow \forall \; x X(x)]. \end{aligned}$$

${\mathsf {PA}}_{\mathsf {2}}$ is categorical, i.e. it has (up to isomorphism) exactly one model ${\mathbb {N}}$. Let now ${\mathsf {PA}}_{\mathsf {2Tr}}$ be any consistent theory of truth for ${\mathsf {PA}}_{\mathsf {2}}$. Since every true sentence of the language of ${\mathsf {PA}}_{\mathsf {2}}$ is true-in-${\mathbb {N}}$, for every sentence $\varphi$ of the language of ${\mathsf {PA}}_{\mathsf {2}}$, every model of ${\mathsf {PA}}_{\mathsf {2Tr}}$ is a model of $\varphi$ only if every model of ${\mathsf {PA}}_{\mathsf {2}}$ is a model of $\varphi$. That is, any consistent theory of truth for ${\mathsf {PA}}_{\mathsf {2}}$ is *ipso facto* semantically conservative.^9^

Consider now the second case. If one’s conception of arithmetic isn’t categorical, it cannot be said to be constituted by a grasp of the standard model ${\mathbb {N}}$. Waxman maintains that it must be exhausted by one’s acceptance of some fixed theory, such as PA. However, Waxman argues, if one’s conception of arithmetic is exhausted by PA, the deflationist can justifiably reject ${\mathsf {PA}}_{\mathsf {Tr}}^+$ in favour of ${\mathsf {PA}}_{\mathsf {Tr}}$, on the grounds that the former, unlike the latter, allows one to prove sentences such as $G_{\mathsf {PA}}$ and Con(PA), and therefore exceeds one’s conception of arithmetic (Waxman 2017, p. 456).^10^ Waxman concludes that, irrespective of whether one’s conception of arithmetic is categorical or axiomatic, ‘[e]ither way, the deflationist is in the clear’ (p. 431). Before turning in Sects. 4–5 to the second horn of Waxman’s argument, a few words on the first horn are in order.

Shapiro (1998, p. 509) already observed that ‘[a]rithmetic truth is semantically conservative over arithmetic, in the sense that any model of second-order arithmetic can be extended to a model of arithmetic-plus-arithmetic-truth’ and that, for this reason, ‘second-order logic may be the salvation for the deflationist’. However, Shapiro correctly points out that establishing the semantic conservativeness of a given theory of truth doesn’t help the deflationist establishing the ‘thinness’ of truth: all it shows is that truth isn’t ‘thicker’ than truth-in-a-second order structure.^11^ But, it might be insisted, such a notion is already ‘thick’. For instance, as Shapiro (1991) points out, ‘a considerable amount of mathematics can be expressed in (pure) second-order languages and, moreover, second-order logic is thoroughly intertwined with set theory’ (Shapiro 1991, p. 97).^12^ In what follows, we focus on the first horn of Waxman’s argument, and on syntactic ways of circumventing the conservativeness argument more generally.

## The implicit commitment thesis

We begin with a preliminary point. Intuitively, if one accepts a mathematical theory *S*, one is also implicitly committed to accepting claims that go over and above *S*. Walter Dean calls this the *implicit commitment thesis*:^13^

(ICT): Anyone who accepts the axioms of a mathematical theory *S* is thereby also committed to accepting various additional statements which are expressible in the language of *S* but which are formally independent of its axioms. (Dean 2015, p. 31)

The schema of local reflection for PA, according to which if PA proves $\varphi$ then $\varphi$, is a case in point:

$$\begin{aligned} {\mathsf {RFN}}_{\mathsf {PA}} \qquad {\mathsf {Prov}}_{\mathsf { PA}}(\ulcorner \varphi \urcorner ) \rightarrow \varphi , \end{aligned}$$

where ${\mathsf {Prov}}_{\mathsf {PA}}$ is a standard provability predicate for PA.

Proponents of ICT typically maintain that, if one accepts PA, one is implicitly committed to its soundness, encoded by ${\mathsf {RFN}}_{\mathsf {PA}}$, and to its consistency, encoded by ${\mathsf {Con(PA)}}$. For instance, Graham Leigh and Leon Horsten have recently argued that

when we are justified in believing a theory, we do not need extra justification for adopting a reflection principle for that theory. In such a situation, we are entitled to adopt a reflection principle without giving additional justification for accepting it. (Leigh and Horsten 2017, p. 211)

However, it is well known that not all instances or ${\mathsf {RFN}}_{\mathsf {PA}}$ are provable in PA: since any theory that proves all instances of ${\mathsf {RFN}}_{\mathsf {PA}}$ also proves ${\mathsf {Con(PA)}}$, PA does not prove all instances of ${\mathsf {Prov}}_{\mathsf {PA}}(\ulcorner \varphi \urcorner ) \rightarrow \varphi$.

Waxman does not deny that the ability to prove ${\mathsf {RFN}}_{\mathsf {PA}}$ is ‘an attractive feature for a theory of truth to possess’ (Waxman 2017, p. 456, fn. 34). But, as he himself points out, it is clearly incompatible with the axiomatic deflationist’s own notion of acceptance, according to which to accept *S* is to accept all and *only* the theorems of *S*. As Waxman puts it, a theory’s ability to prove ${\mathsf {RFN}}_{\mathsf {PA}}$

presupposes the falsity of the axiomatic conception of arithmetic, for it requires the acceptance of truths in the language of arithmetic that do not follow (in the relevant sense) from its axioms. (Waxman 2017, p. 456, fn. 34)

Intuitive though it may seem, the implicit commitment thesis is precluded to the axiomatic deflationist.

## Two problems

According to the axiomatic conception of arithmetic, ‘our best understanding of arithmetic consists in (and is exhausted by) the proof-theoretic consequences of a particular set of axioms, namely, first-order PA’ (Waxman 2017, p. 447). A conservativeness requirement seemingly follows from such a conception: if one’s conception of arithmetic is exhausted by PA, one shouldn’t accept arithmetical sentences that are not derivable in PA. More precisely, the axiomatic deflationist should be agnostic about sentences such as $G_{\mathsf {PA}}$, i.e. she should neither accept nor reject them. Non-conservative theories of truth are therefore to be rejected, on the grounds that they prove arithmetical sentences one doesn’t accept. In particular, if one’s understanding of arithmetic is exhausted by PA, one should accept ${\mathsf {PA}}_{\mathsf {Tr}}$ as opposed to ${\mathsf {PA}}_{\mathsf {Tr}}^+$, since ${\mathsf {PA}}_{\mathsf {Tr}}^+$ allows one to prove $G_{\mathsf {PA}}$ and $G_{\mathsf {PA}}$ is not provable in PA.

Axiomatic deflationists face at least two problems, however. The first is that $G_{\mathsf {PA}}$ appears to be a standard piece of mathematical knowledge—one that should be included in any viable account of mathematics. For instance, here is Andrzej Mostowski:

We see that the [Gödel] sentence $G_ {\mathsf {PA}}$ is intuitively obvious and does not represent any difficult mathematical problem the solution of which would surpass our mathematical knowledge. (Mostowski 1952, p. 107, cited in Dean (2015))

Knowledge of sentences like $G_ {\mathsf {PA}}$ is ‘intuitively obvious’. Yet, it seems necessarily precluded to the axiomatic deflationist, who must thereby place herself outside the community of mathematical practitioners.

The second problem is that, even though the axiomatic deflationist demurs from accepting $G_ {\mathsf {PA}}$, her argument against non-conservative theories *assumes*$G_ {\mathsf {PA}}$. More precisely, the justification Waxman attributes to the axiomatic deflationist for demurring from accepting sentences like $G_{\mathsf {PA}}$, and non-conservative theories of truth more generally, is *self-undermining*. Let us explain.

According to Waxman,

[the] deflationist has a *principled reason* to accept ${\mathsf {PA}}_{\mathsf {Tr}}$ [...] and in particular to demur from accepting ${\mathsf {PA}}_{\mathsf {Tr}}^+$. The reason for resisting the move to ${\mathsf {PA}}_{\mathsf {Tr}}^+$ more or less falls out of the axiomatic characterization of arithmetic: extending induction to cover sentences containing [Tr] allows the derivation of $[G_{\mathsf {PA}}]$—a sentence that is (on this view) not licensed by the background understanding of arithmetic, *since it is not derivable from the axioms*. (Waxman 2017, p. 456; emphases added)

However, Waxman’s reason for not ‘requiring the derivation of [$G_{\mathsf {PA}}$]’ is that $G_{\mathsf {PA}}$ ‘is not derivable from the axioms [of PA]’ (Waxman 2017, p. 431). Yet this very sentence, i.e. ‘$G_{\mathsf {PA}}$ is not provable in ${\mathsf {PA}}$’, is *provably equivalent* to $G_{\mathsf {PA}}$ in PA. More specifically, ‘$G_{\mathsf {PA}}$ is not provable in ${\mathsf {PA}}$’ is expressed in ${\mathsf {PA}}$ itself as $\lnot {\mathsf { Prov}}_{\mathsf {PA}}(\ulcorner G_{\mathsf {PA}}\urcorner )$, which in PA is provably equivalent to $G_{\mathsf {PA}}$. Thus, the claim that $G_{\mathsf {PA}}$ isn’t derivable in PA*requires accepting*$G_{\mathsf {PA}}$*itself*, and hence cannot be accepted by someone whose conception of arithmetic is exhausted by ${\mathsf {PA}}$. In conclusion, the axiomatic deflationist’s justification for not accepting theories that prove sentences she cannot accept *requires accepting these very sentences*.

## Three unsuccessful strategies

How can the axiomatic deflationist respond to the above problems? We see at least three avenues of reply, which we explore in turn.

### Silence

In response to the objection from the self-undermining nature of her demurral from accepting non-conservative theories of truth, the axiomatic deflationist might just bite the bullet, and simply refrain from offering a principled reason for not accepting sentences like $G_{\mathsf {PA}}$. That is, she might insist that accepting a theory *S* while simply demurring from accepting its Gödel sentence $G_{S}$ (as well as theories that are non-conservative over *S*) results in a perfectly stable position—if not one she is able to explicitly justify.

This seems deeply unsatisfactory, though, for at least two reasons. First, when presented with ${\mathsf {PA}}_{\mathsf {Tr}}$ and ${\mathsf {PA}}_{\mathsf {Tr}}^+$ as candidate theories of truth for PA, the axiomatic deflationist who adopts this line of reply is forced to choose blindly: in order to motivate her choice, she cannot offer grounds that are in line with her conservative conception of truth. To be sure, such a deflationist will prefer ${\mathsf {PA}}_{\mathsf {Tr}}$ over ${\mathsf {PA}}_{\mathsf {Tr}}^+$. However, she will do so for reasons that are in principle not accessible to her, as required by her ‘strategy of silence’. Second, this response does not address the first problem mentioned above, that sentences like $G_{\mathsf {PA}}$ are simply common mathematical knowledge.

### Iasaacson’s thesis and Tennant’s strategy

In response to the two problems mentioned in Sect. 4, the axiomatic deflationist might retort that she does have a reason to demur from accepting non-conservative theories of truth while at the same time accepting $G_{\mathsf {PA}}$. It is just that such a reason is *not part of her conception of arithmetic*. More precisely, the axiomatic deflationist might follow Daniel Isaacson (1987, 1992) and distinguish between two kinds of ${\mathcal {L}}_{\mathsf {PA}}$-sentences. On one hand, there are sentences that are ‘directly perceivable as true from our grasp of the fundamental nature and structure of the natural numbers’ (Isaacson 1992, p. 95), which extensionally coincide with the set of PA’s theorems. On the other hand, there are sentences of ${\mathcal {L}}_{\mathsf {PA}}$ that are unprovable in PA, such as $G_{\mathsf {PA}}$, that are ‘shown to be true by an argument in terms of truths concerning some higher-order notion’ (Isaacson 1987, p. 220). The axiomatic deflationist might then endorse *Isaacson’s Thesis*, viz. the idea that the set of arithmetical truths is to be identified with the set of theorems of PA, and that the proofs of true ${\mathcal {L}}_{\mathsf {PA}}$-sentences that are unprovable in PA require ‘ideas that go beyond those that are required in understanding PA’ (Smith 2008, p. 1). Armed with such a thesis, she might insist that any proof of the non-conservativeness of ${\mathsf {PA}}_{\mathsf {Tr}}^+$ over PA should be seen as *extra-arithmetical*, so that the assertion of sentences of ${\mathcal {L}}_{\mathsf {PA}}$ that are unprovable in PA need not exceed her conception of *arithmetic*.

In a similar vein, the deflationist might appeal to a strategy advocated by Neil Tennant (2002) to argue that the grounds for accepting $G_{\mathsf {PA}}$ need not be truth-theoretic. According to Tennant, the deflationist can adopt a syntactically conservative theory of truth while at the same time proving sentences such as $G_{\mathsf {PA}}$ by means of non-truth-theoretic principles such as the local reflection principle for PA:

$$\begin{aligned} {\mathsf {RFN}}_{\mathsf {PA}} \qquad {\mathsf {Prov}}_{\mathsf {PA}}(\ulcorner \varphi \urcorner ) \rightarrow \varphi \end{aligned}$$

This is how, in Tennant’s view, the deflationist can meet the challenge, originally raised by Ketland (1999), to account for our knowledge of $G_{\mathsf {PA}}$, without thereby validating a non-conservative conception of truth.^14^ Given the distinction between arithmetical and extra-arithmetical truths of ${\mathcal {L}}_{\mathsf {PA}}$ provided by Isaacson’s Thesis, the axiomatic deflationist might employ ${\mathsf {RFN}}_{\mathsf {PA}}$ to prove standard pieces of mathematical knowledge such as $G_{\mathsf {PA}}$ and ${\mathsf {Con(PA)}}$, without thereby exceeding her conception of arithmetic as given by PA.

Unfortunately, though, neither Isaacson’s Thesis nor Tennant’s Strategy help the axiomatic deflationist addressing the problems mentioned in Sect. 4. For if the axiomatic deflationist ought to accept $G_{\mathsf {PA}}$ on the grounds that it is a standard piece of mathematical knowledge, she ought to arguably also accept *other* standard pieces of mathematical knowledge. However, this leads her to accept mathematical theories that re-introduce a non-conservative notion of arithmetical truth. More specifically, the axiomatic deflationist who wishes to retain common mathematical knowledge arguably ought to accept ACA, a subsystem of second-order arithmetic that can be seen as a formalisation of a fragment of analysis.^15^ But here lies the problem. ACA and ${\mathsf {PA}}_{\mathsf {Tr}}^+$ are intertranslatable, in a sense made precise by the following result:

#### **Theorem 3**

(Halbach 2014, Theorem 8.42, pp. 108–115) *The systems*${\mathsf {PA}}_{\mathsf {Tr}}^+$*and*ACA*are proof-theoretically equivalent*. *More precisely*, ${\mathsf {PA}}_{\mathsf {Tr}}^+$’*s truth predicate can be defined in*ACA*and there is a relative interpretation of*ACA*in*${\mathsf {PA}}_{\mathsf {Tr}}^+$*that does not reinterpret arithmetical expressions*.

For our purposes, the crucial part of the theorem is the definability of ${\mathsf {PA}}_{\mathsf {Tr}}^+$’s truth predicate in ACA. This shows that, if one accepts ACA, then one *ipso facto* also accepts the truth predicate of ${\mathsf {PA}}_{\mathsf {Tr}}^+$, since that truth predicate is implicitly contained, and can be explicitly defined, in ACA. However, recall, ${\mathsf {PA}}_{\mathsf {Tr}}^+$’s truth predicate is arithmetically non-conservative, and hence unacceptable by (conservative) deflationist lights. Thus, the conservative deflationist who wishes to retain common mathematical knowledge must reject not only ${\mathsf {PA}}_{\mathsf {Tr}}^+$, but also, implausibly, ACA together with the standard fragment of mathematics it encodes.

In conclusion, the axiomatic deflationist might appeal to Isaacson’s Thesis to retain knowledge of sentences such as ${G}_{\mathsf {PA}}$ while at the same time rejecting non-conservative notions of truth. Furthermore, she might invoke Tennant’s Strategy to show how, more precisely, such sentences might be established, without making use of a non-conservative truth predicate. However, this is not enough: since an arithmetically non-conservative truth predicate is definable within ACA, the conservative deflationist must either adopt a non-conservative conception of arithmetical truth, or give up standard pieces of mathematical knowledge such as the fragment of analysis encoded by ACA.

### Conservative deflationism beyond PA?

The deflationist might object at this point that she is out to defend syntactically conservative *mathematical* truth—not just conservative *arithmetical* truth. That is, she might consider a strong mathematical theory representing all or most of our mathematical knowledge, call it *M*, and demand that truth be conservative over *it*. To be sure, sentences such as Con(*M*), a sentence expressing *M*’s consistency, or $G_M$, a Gödel sentence for the theory *M*, would still fall out of the deflationist’s conception of mathematics. However, the deflationist might reasonably argue that this need not be a problem. In the case of *M*, agnosticism about *M*’s consistency statement and its Gödel sentence seems justified, on the grounds that Con(*M*) and $G_M$ (and more generally sentences independent from *M*), are *not* common mathematical knowledge.

George Boolos (1990) famously voiced a similar view about ZF:

I suggest that we do not know that we are not in the same situation vis-à-vis ZF that Frege was in with respect to naive set theory [...] before receiving [...] the famous letter from Russell, showing the derivability in his system of Russell’s paradox. It is, I believe, a mistake to think that we can see that mathematics as a whole is consistent, a mistake possibly fostered by our ability to see the consistency of certain of its parts. (Boolos 1990, p. 390)

Even if one thinks that the consistency of ZF is quite safe, the spirit of Boolos’s remark still stands: if *M* is sufficiently powerful and complex, it is at least doubtful that we can be in a position to know *M*’s consistency.

Nevertheless, considering strong mathematical theories does not help the axiomatic deflationist with the problem at hand: she still cannot coherently justify her demurral from accepting non-conservative theories of truth over *M*. For suppose the deflationist’s conception of mathematics is exhausted by her acceptance of *M*. Suppose moreover she is given the choice between two theories of truth over *M*: a conservative one, call it $M_{\mathsf {Tr}}$, and a non-conservative one, call it $M_{\mathsf {Tr}}^+$. How can she justify her choice of $M_{\mathsf {Tr}}$ over $M_{\mathsf {Tr}}^+$? As before, such a deflationist cannot simply reject $M_{\mathsf {Tr}}^+$ on the grounds that it proves $G_M$, on pain of presupposing $G_M$ itself.

Could the problem be solved by appealing, once again, to an analogue of Isaacson’s Thesis for *M*? More precisely, could an analogue of Isaacson’s Thesis be invoked to claim that $G_M$ is *non-mathematical*? Perhaps, but we see two difficulties with this suggestion. For one thing, it is unclear whether an analogue of Isaacson’s Thesis for *M* is available. Leon Horsten (2001, p. 173) defends an analogue of the thesis for ZFC, to the effect that ‘the collection of mathematical truths is identical with the set of theorems of ZFC’. However, as far as we can see, Horsten’s thesis has been effectively criticised by Luca Incurvati (2008). For another, it is unclear what kind of non-mathematical knowledge sentences such as Con(*M*) and $G_M$ could possibly represent. In the case of arithmetic it is at least plausible to claim that certain sentences of ${\mathcal {L}}_{\mathsf {PA}}$ are extra-arithmetical *and yet still mathematical*, on the grounds that they are not about natural numbers, but about meta-theoretic notions such as provability. By contrast, in the case of *M* it is unclear what kind of subject matter sentences such as Con(*M*) and $G_M$ could possibly have.

Some recent results by Fujimoto (2017) might be thought to tell against the argument just given. According to Fujimoto,

the appropriate formal setting for evaluating the adequacy or inadequacy of the conservativeness argument is provided not by theories of truth over arithmetic but by those over much ‘richer’ subject matters such as set theory. (Fujimoto 2017, p. 4)

Theories based on arithmetic differ from theories of truth based on set theory in an important respect: unlike arithmetic, ‘set theory intrinsically contains a theory of syntax and is ‘rich’ enough to implement substantial meta-mathematics on the basis of it’ (p. 17). As a result, typed theories of compositional truth with unrestricted induction over set theory, i.e. set-theoretical analogues of ${\mathsf {PA}}_{\mathsf {Tr}}^+$, are conservative over their base theories (Fujimoto 2017, Theorems 1 and 2, pp. 18–19). Axiomatic deflationists might appeal to results such as these in order to claim that deflationary theories of truth can be both conservative and adequate, in spite of the conservativeness argument.

However, the objection fails to convince, for at least two reasons. First, not all theories of truth over strong base theories are conservative. Fujimoto (2017, Theorem 3, p. 20) himself provides an example of a non-conservative theory of truth over a strong base theory. Second, the adoption of a conservative theory of truth still won’t help the axiomatic deflationist offering a non-self-undermining reason to demur from accepting non-conservative theories in favour of conservative ones: this would require, again, accepting sentences not provable in the base theories in question.

In summary, the axiomatic deflationist is free to identify her conception of mathematics with the adoption of a strong mathematical theory, thereby retaining standard pieces of mathematical knowledge such as $G_{\mathsf {PA}}$, fragments of analysis, and beyond. However, our second objection still applies. As before, the axiomatic deflationist is not in a position to justify her demurral from accepting sentences such as Con(*M*) and $G_M$ (and hence non-conservative theories of truth) because they exceed their conception of mathematics.

## Concluding remarks

Both Waxman and Tennant point to the existence of syntactic ways out of the conservativeness argument. In Waxman’s view, the axiomatic deflationist can coherently reject non-conservative theories of truth on the grounds that they exceed her conception of arithmetic. As for Tennant, he suggests that standard pieces of mathematical knowledge such as $G_{\mathsf {PA}}$ and ${\mathsf {Con}}({\mathsf {PA}})$ can be recaptured via proof-theoretic means, without resorting to truth-theoretic resources. However, we have argued that the axiomatic deflationist faces at least two difficulties. First, even if she can come to know $G_{\mathsf {PA}}$ and ${\mathsf {Con}}({\mathsf {PA}})$, she still seems unable to recover other standard pieces of mathematical knowledge, such as the fragment of analysis encoded by ACA, on pain of being committed to a non-conservative conception of arithmetical truth. Second, the deflationist does not seem to be in a position to explain why sentences such as $G_{\mathsf {PA}}$ and ${\mathsf {Con}}({\mathsf {PA}})$, and non-conservative theories of truth more generally, are not to be accepted. Even if the former problem can be addressed by restricting one’s attention to strong base theories, and requiring one’s theory of truth to be conservative over them, the second problem is not easily solved. As soon as the axiomatic deflationist explicitly articulates her reason for demurring from accepting sentences that are not provable in the theory the acceptance of which she takes to constitute her conception of mathematics, she thereby accepts sentences that exceed such a conception.

## Footnotes

1 See also Picollo and Schindler (2017, Sect. 6).

2 The question whether deflationary truth should be conservative has been debated in a number of places. See e.g. Shapiro (1998), Azzouni (1999), Ketland (1999, 2005, 2010), Field (1999), Halbach (2001), Tennant (2002, 2005), Horsten (2009), Cieśliński (2010, 2015) and Waxman (2017).

3 For convenience, we talk of the Gödel sentence for PA, even though several such sentences can be constructed. For general background on PA, $G_{\mathsf {PA}}$, and Con(PA) see e.g. Smorinski (1977) and Kaye (1991).

4 In Waxman’s reconstruction, the categorical deflationist resorts to second-order arithmetic. Going beyond arithmetic, we also notice that a notion of consequence that exceeds first-order logical consequence is required for the quasi-categoricity or categoricity of stronger mathematical theories such as (second-order versions of) ZFC or ZFC with *Urelemente*. We thank an anonymous referee for drawing our attention on this point.

5 The view can be traced back to Hilbert [see e.g. Hilbert (1959) and the letters to Frege of December 29, 1899 and September 22, 1900 (Hilbert 1935)] and still counts a number of supporters [see e.g. Curry (1951), Robinson (1969), Detlefsen (1986), Tennant (1997), Gabbay (2010) and Weir (2010)]. As we will see in Sect. 5.2, the position articulated in Isaacson (1987) can also be added to the list: if correct, his arguments can be seen as establishing that all of the true arithmetical sentences that are properly about natural numbers are derivable in Peano Arithmetic, in spite of the incompleteness theorems.

6 The view that deflationism ought to be dissociated from the conservativeness requirement is by no means new. See e.g. Halbach (2001), Horsten (2012, Sect. 7.5), Cieśliński (2015) and Galinon (2015).

7 More precisely, the Tarskian compositional axioms for conjunction, conditional, and universal quantifier are as follows:

$$\begin{aligned} (\wedge )\quad&\forall x \forall y ({\mathsf { Sent}}_{\mathcal {L}_{\mathsf {PA}}}(x {\mathop {\wedge }\limits _{\cdot }} y) \rightarrow ({\mathsf { Tr}}(x {\mathop {\wedge }\limits _{\cdot }} y) \leftrightarrow {\mathsf {Tr}}(x) \wedge {\mathsf {Tr}}(y)))\\ (\rightarrow )\quad&\forall x \forall y ( {\mathsf {Sent}}_{\mathcal {L}_{\mathsf {PA}}}(x {\mathop {\rightarrow }\limits _{\cdot }} y) \rightarrow ({\mathsf {Tr}}(x {\mathop {\rightarrow }\limits _{\cdot }} y) \leftrightarrow {\mathsf {Tr}}(x) \rightarrow {\mathsf {Tr}}(y)))\\ (\forall )\quad &\forall x \forall y ({\mathsf {Sent}}_{\mathcal {L}_{\mathsf {PA}}}({\mathop {\forall }\limits _{\cdot }}xy) ({\mathsf {Tr}}({\mathop {\forall }\limits _{\cdot }}xy) \leftrightarrow \forall t{\mathsf {Tr}}(x[t/y]))) \end{aligned}$$

${\mathsf {Sent}}_{\mathcal {L}_{\mathsf {PA}}}$ is an arithmetically definable predicate that represents the sentences of ${\mathcal {L}}_{\mathsf {PA}}$ in ${\mathcal {L}}_{\mathsf {PA}}$, while dotted logical operators (e.g. ${\mathop {\wedge }\limits _{\cdot }}$) indicate their representation in ${\mathcal {L}}_{\mathsf {PA}}$.

8 For a detailed analysis of ${\mathsf {PA}}_{\mathsf {Tr}}$ and ${\mathsf {PA}}_{\mathsf {Tr}}^+$, see Halbach (2014, Ch. 8)

9 As Waxman acknowledges, this fact had long been pointed out by Shapiro (1998, p. 509 and ff.). To be sure, suitable choices of ${\mathsf {PA}}_{\mathsf {2Tr}}$ may yield a non-conservative extension of ${\mathsf {PA}}_{\mathsf {2}}$ in the syntactic sense. However, the semantic deflationist can reasonably maintain that this should be no cause of concern, since one’s notion of consequence is then given by the full (much more powerful) second-order consequence relation (see Waxman 2017, p. 457).

10 As Waxman points out, Azzouni (1999) already argues in favour of ${\mathsf {PA}}_{\mathsf {Tr}}$ on precisely these grounds.

11 We thank [redacted] for a very helpful discussion on this point.

12 In a similar vein, Cezary Cieśliński has recently argued that ‘describ[ing] arithmetical truth—truth *simpliciter*—as truth is some chosen (intended) model of arithmetic, while treating the last notion as indispensable and primary’ is incompatible with the deflationary doctrine that ‘truth *simpliciter* is fully characterised by nothing other than [$\ldots$] basic truth axioms’ (Cieśliński 2015, p. 73).

13 Versions of the thesis have been famously proposed and explored by Solomon Feferman in a number of articles [see e.g. Feferman (1962), Feferman (1991); see also Franzen (2004)]. It is sometimes objected that ICT is incompatible with certain foundational positions in the philosophy of mathematics. For a recent discussion, see e.g. Dean (2015, p. 32) and Nicolai and Piazza (2017).

14 For more discussion on this point, see also Ketland (2005), Tennant (2005), Incurvati (2009), Cieśliński (2010) and Ketland (2010).

15 ACA is a non-categorical second-order theory in which the comprehension axiom is restricted to arithmetically definable sets. See e.g. Friedman and Simpson (2000, p. 128) and Halbach (2014, p. 94).
